# Neural Crest-Derived Stem Cells (NCSCs) Obtained from Dental-Related Stem Cells (DRSCs): A Literature Review on Current Knowledge and Directions toward Translational Applications

**DOI:** 10.3390/ijms23052714

**Published:** 2022-02-28

**Authors:** Oscar O. Solis-Castro, Marcelo N. Rivolta, Fiona M. Boissonade

**Affiliations:** 1 School of Clinical Dentistry, University of Sheffield, Sheffield S10 2TA, UK; o.soliscastro@sheffield.ac.uk; 2 The Neuroscience Institute, University of Sheffield, Sheffield S10 2TN, UK; m.n.rivolta@sheffield.ac.uk; 3 Centre for Stem Cell Biology, Department of Biomedical Science, University of Sheffield, Sheffield S10 2TN, UK

**Keywords:** dental-related stem cells, neural crest-derived stem cells, ectomesenchymal, adult stem cells, regenerative medicine

## Abstract

Evidence from dental-related stem cells (DRSCs) suggests an enhanced potential for ectodermal lineage differentiation due to their neural crest origin. Growing evidence that DRSC cultures can produce cells with a neural crest-derived stem cell (NCSC)-like phenotype supports their potential for future therapeutic approaches for neurodegenerative diseases and nerve injuries. However, most of the evidence is limited to the characterization of DRSCs as NCSCs by detecting the expression of neural crest markers. Only a few studies have provided proof of concept of an improved neuro-glial differentiation or direct applicability in relevant models. In addition, a current problem is that several of the existing protocols do not meet manufacturing standards for transferability to a clinical scenario. This review describes the current protocols to obtain NCSCs from DRSCs and their characterization. Also, it provides important considerations from previous work where DRSCs were established and characterized as mesenchymal stromal cells but studied for their neuro-glial differentiation potential. The therapeutic advancement of DRSCs would depend on establishing protocols that can yield a neural crest-like phenotype efficiently, using appropriate manufacturing standards and testing them in relevant models of disease or injury. Achieving these conditions could then facilitate and validate the therapeutic potential of DRSC-NCSCs in regenerative therapies.

## 1. Introduction

The subgroup of adult stem cells defined in vitro as mesenchymal stromal cells (MSCs), are proposed as an ideal source of cells with therapeutic potential because of their multipotent differentiation, potential availability throughout life, and their possible use for autologous cell therapies. MSCs have been extracted from several tissues such as bone marrow, adipose tissue, cartilage, tendon, gut, umbilical cord blood, salivary glands and dental pulp [1,2,3,4,5]. Nevertheless, the expanding field of stem cell research suggests intrinsic differences among the different sources of MSCs. For instance, dental-related stem cells (DRSCs)—derived from dental pulp, gingiva and periodontal ligament—have been extensively characterized as MSCs. However, they have also been proposed recently as a source of cells phenotypically resembling neural crest cells (neural crest-derived stem cells or NCSCs), given their embryological neural crest origin. It is believed that adult stem cells derived from the neural crest could be more efficient in deriving ectodermal lineages such as neurons and glia cells. The neuronal regenerative potential of DRSCs has been reviewed elsewhere [6,7,8].

The literature reviewed here introduces and describes how the neural crest developmental origin of DRSCs is used to obtain NCSCs. We will discuss the main strategies for obtaining a NCSC phenotype, their limitations, and the proposed advantages of using DRSC-NCSCs for the generation of ectodermal lineages, as opposed to DRSCs characterized as MSCs. We have limited our discussion to work that clearly describes and characterizes DRSCs as having a neural crest-like phenotype, excluding previous reports in which similar conditions may have been used, but where DRSCs were not defined molecularly as NCSCs.

## 2. The Neural Crest and Its Adult Stem Cell Derivatives

The neural crest is a transient embryonic cell population that begins its formation at the interface between the neural and non-neural ectoderm (i.e., the neural plate border). Toward the time of dorsal tube closure, the neural crest cells (NCCs) begin their migration from the neural plate border by triggering the epithelial-to-mesenchymal transition (EMT), followed by cell fate commitment and establishing a variety of organs and tissues. The neural crest is said to be the fourth germ layer because of its pluripotency and ability to form a range of cell fates, such as neurons, glia, cardiac muscle, osteoblasts, chondrocytes and melanocytes [9].

Recombinant technologies available in animal models have helped to elucidate several niches of adult stem cells derived from the neural crest—hence, neural crest-derived stem cells. Murine models utilising a green fluorescent protein (GFP) signal, driven by the expression of Wingless-related integration site-1 (WNT1) and P0, has allowed the tracing of NCCs into adult niches such as bone marrow, dorsal root ganglia, whisker pads and iris stroma [10,11,12]. Furthermore, cultured neural crest-derived stem cells have been shown to express neural crest-related markers (e.g., Nes, Sox9, p75, Slug, Snail, Sox10, Pax3, Twist1), and to differentiate in vitro into endoderm (e.g., insulin-producing cells [13]) mesoderm (e.g., osteocytes, adipocytes, chondrocytes, smooth muscle [10,14]) and ectoderm (e.g., Schwann cells and neurons [10,14,15,16]) lineages. NCCs contribute greatly to the development of the peripheral nervous system. This suggests the potential of NCSCs found in adult tissues to be used in the treatment of neurodegenerative disorders. Indeed, neuroglial differentiation from rodent-derived tissues has been shown for palatal cells, bulge follicle cells and iris stroma, among others [11,17,18].

It is very difficult to trace embryonic NCCs to determine the origin of resident stem cells in humans as has been done in animal models. However, it is important to translate results from animal findings into man in order to progress their proposed clinical applications. Assumptions of a developmental analogy can be supported by the expression of neural crest markers and the ability to culture cells in vitro with neural crest characteristics. Current work has begun to characterize NCSCs derived from human tissues including the palate, inferior turbinate, bulge hair follicle, and from DRSCs [18,19,20,21].

## 3. Dental-Related Sources of Human Neural Crest-Derived Stem Cells

**Figure 1:**
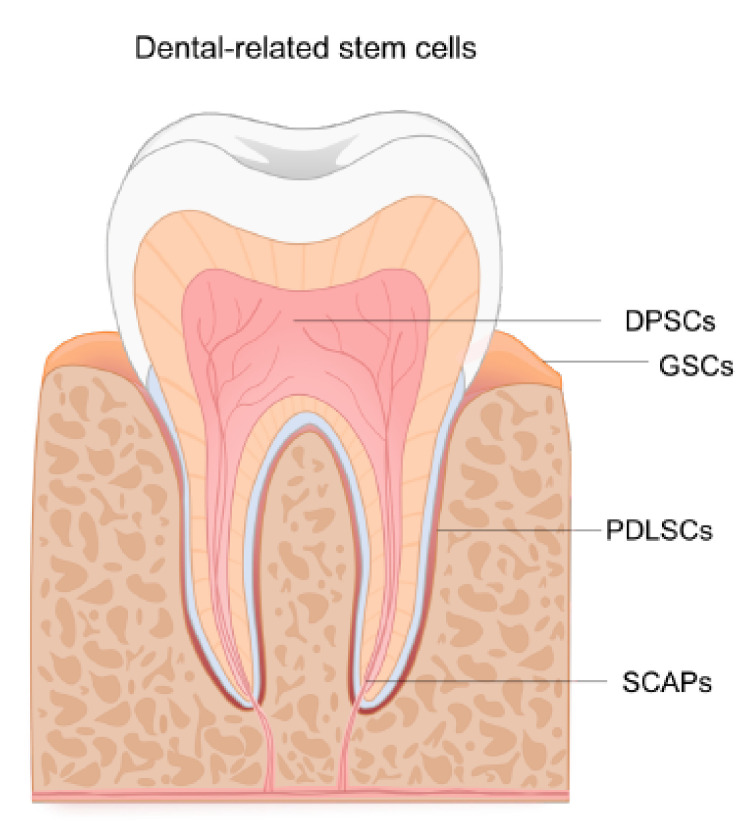
Sources of stem cells in adult dental tissues. Stem cells can be found and isolated from dental tissues including dental pulp stem cells (DPSCs), gingiva stem cells (GSCs), periodontal ligament stem cells (PDLSCs) and stem cells from apical pulp (SCAPs). This diagram does not include stem cells from developing teeth.

There are a number of advantages associated with using dental tissues as a potential source of stem cells. For instance, the source of DRSCs is accessible through routine dental procedures where samples and/or biopsies can be taken from surplus tissue, commonly discarded after the procedures (Figure 1), thus making them ideal for autologous cell therapies. However, it would be necessary to extend the current knowledge of dental tissues as a potential source of human NCSCs to be used in regenerative medicine and in particular for neural-related issues. The current interest and proposed advantages of obtaining NCSCs have resulted in the growth and characterization of DRSCs for their neural crest-like phenotype, which is described below (see also Table 1 for a summary).

### 3.1. NCSCs Derived from Gingiva

Several previous studies have reported the gingiva as a source of stem cells [22,23,24]. When grown as a monolayer in serum-containing medium, neural crest characterization of human gingival fibroblast cultures (GF) and GF-derived clone cultures (gingival stem cells; GSC), showed mRNA expression of *SNAI1*, *TWIST1*, *SOX9*, *NES*, *FOXD3* and *PAX3* [25]. The authors demonstrated that further growth of GFs as neurospheres resulted in the presence of Cx43, TUBIII, COL1 VIM and FN (shown by immunocytochemistry [ICC]), and presented higher mRNA levels of *NES*, *Tenancin-C* and *SOX9* than did their GF monolayer counterpart [25]. The results suggest an initial basal level of neural crest identity, that can be increased by modifying the culture conditions to non-adherent cultures.

Additionally, Zhang et al. [26] described NCSCs from human gingiva obtained by different approaches: (i) spontaneous detached spheres formed from monolayer cultures; (ii) 3D spheroids grown in ultra-low attachment plates; and (iii) by the use of small molecules for WNT activation coupled with TGF-β inhibition in monolayer cultures. The latter condition resulted in cells with epithelial morphology and an enhanced expression of FOXD3, SLUG and SOX9 proteins, while also producing a gradual decrease of the MSC markers CD90, CD44, CD29 and CD73. The authors also provided relevant evidence for their regenerative capability in a rat model of facial nerve regeneration. Insertion of NCSCs into nerve conduits produced higher expression of S100β, TUBIII, NF and GAP-43 in regenerated nerves, compared with insertion of their MSC counterpart, together with more myelination and larger compound action potentials [26]. This evidence not only represents one of the few functional, relevant results from DRSCs, but also highlights the contrast of MSCs vs. NCSCs in terms of their regenerative potential.

**Table 1:** Summary of human DRSCs described as NCSCs. Reported methods for growth and characterization from DRSCs described as NCSCs.

Origin	Method	Medium Components	Characterization	Functional Assay	Ref
Gingiva	Self-induced spheres and sphere-derived monolayer cultures From monolayer cultures grown in standard FBS-containing medium	NCSC medium (PLO/LAM coating): DMEM/F12, neurobasal medium, human bFGF (20 ng/mL), human EGF (20 ng/mL), βMe (55 µM), N2 (1%), B27 (1%)	*ICC:* P75, NES, SOX9, SNAIL1 *RT-qPCR*: *SOX9*, *FOXD3*, *SLUG*, *SNAIL1*	Nerve conduits in rats: functional recovery	[26]
	Small molecules (monolayer) NCSCs with WNT activation and TGFβ inhibition	Supplemented medium: NCSCs medium + CHIR99021 (2.5 µM), SB43152 (5 µM)	*Flow cytometry:* Loss: CD29, CD44, CD73, CD90 Gain: P75	None	
	Neurospheres From monolayer cultures grown in standard FBS-containing medium	Neurosphere medium: DMEM/F12, L-Glut (2 mM), EGF (20 ng/mL), FGF2 (40 ng/mL), B27 (2%)	Monolayer and neurospheres: *RT-qPCR*: *NES*, *PAX3*, *TWIST1*, *SNAIL1*, *FOXD3* AND *SOX9* Neurospheres: *ICC*: VIM, NES, COL1, FN	In vitro neural differentiation	[25]
Periodontal ligament	EGF/FGF2 (monolayer) From monolayer cultures grown in standard FBS-containing medium	a-MEM, FBS (10%), chicken embryo extract (2%), transferrin (10 mg/mL), hydrocortisone (0.1 mg/mL), glucagon (0.01 ng/mL), insulin (1 ng/mL), triiodothyronine (0.4 ng/mL), EGF (0.1 ng/mL), FGF2 (1 ng/mL), pen/strep (100 U/100 µg/mL)	NCSC conditions: *ICC*: P75, HNK-1, NES *RT-PCR*: *NES*, β*TUBIII*, *NFM*, *MAP2*, *PER*, *P0*, *GFAP*, *aSMA.*	None	[27]
	Direct isolation Connexin 43^+ve^ magnetic cell sorting	DMEM 10% FBS	CX43^+ve^ population: *ICC:* NANOG, SOX2, SOX10, OCT4, NES, P75	Teratoma formation as pluripotency test	[28]
	Small molecules (monolayer) From monolayer cultures grown in a defined MSC medium and changed to a NCSCm	MESENDEM (FN-coating): Optimem, ITS (1×), βME (50 µM), Glutamax (1×), FGF2 (5 ng/mL), EGF (10 ng/mL), Anti Anti (1×), 0.5, 1, 2, 10% FBS NCSCm (FN-coating): DMEM/F12, N2 (1×) B27 (1×), FGF2 (10 ng/mL), EGF (10 ng/mL), BIO (2 µM), REPSOX (1 µM)	NCSCs medium: *Flow cytometry:* increase in P75 and HNK1, E-CAD *RT-PCR*: *OCT4*, *SOX2*, *MYC*, *CDH1*, *ZEB*, *SOX10*	None	[29]
Human exfoliated deciduous teeth	Small molecules (monolayer) From monolayer cultures grown in a defined MSC medium and changed to a NCSCm	DentEpiMesMed (FN-coating): DMEM/F12, N2 (1×) ascorbic acid (100 µg/mL), βME (50 µM), Glutamax (1×), FGF2 (2.5 ng/mL), EGF (10 ng/mL) IGF (10 ng/mL), MEM AA (1×), Anti Anti (1×), 1 or 2% FBS NCSCm (FN-coating): DentEpiMesMed + BIO (1 µM), REPSOX (5 µM)	NCSC medium: *Flow cytometry*: Upregulation of P75 and E-cad, downregulation of CD73 *ICC*: Upregulation of SOX10 *RT-qPCR:* upregulation of *SOX10*, *ZO-1*, *E-cadherin*	In vitro neural differentiation (from defined MSC culture, but not from NCSCm cultures)	[30]
Apical pulp	Monolayer: Cultures grown in standard FBS-containing medium	DMEM, 10% FBS, L-Glut (2 mM)	*RT-PCR: SNAIL1*, *SNAIL2*, *SOX9*, *TWIST1*, *MSX2* and *DLX6* *Flow cytometry:* STRO-1 negative	In vitro neural differentiation	[31]
	Neurospheres: Initial growth in standard FBS-containing medium and induced neurosphere formation	Neurosphere medium: DMEM/F12, FGF2 (20 ng/mL), EGF (20 ng/mL), N2	Neurospheres: *ICC:* NES, MSI1, P75 *RT-PCR: NES*, *MSI1*, *Slug*, *Snail*, *P75*	In vitro neural differentiation	[32]
	Neurosphere Initial growth in standard FBS-containing medium and induced neurosphere formation	Neurosphere medium (Matrigel coating after first passage): DMEM/F12, FGF2 (20 ng/mL), EGF (20 ng/mL), N2, B27	*ICC:* HNK-1 and P75	In vitro odontogenic differentiation	[33]
Dental follicle	Monolayer: Cultures grown in standard FBS-containing medium	DMEM/F12, 20% FBS, ascorbic acid (100 µg/mL), L-Glut (2 mM)	*ICC: HNK-1*, *NES*, *P75*, *SOX2*	None	[34]
Dental pulp	EGF/FGF2 (monolayer): Dental pulp cells outgrown from explants	Neurobasal medium, B27 without vitamin A, FGF2 (20 ng/mL), EGF (20 ng/mL), insulin (2.5 µM), L-Glut (2 mM), neuregulin-β1 (10 nM)	NCSC explants: *ICC:* P75, NES, SOX10	Schwann cell, osteogenic and melanocytic differentiation	[35]
	Neurospheres: From monolayer cultures grown in standard FBS-containing medium	DMEM, 10% FBS, L-ascorbic acid (100 µM)	*Flow cytometry:* CD34 negative *ICC:* P75, NES, c-KIT, MART-1	In vitro mesenchymal lineage differentiation	[36]
	Direct isolation + neurosphere: STRO-1^+ve^, c-KIT^+ve^, CD34^+ve^ magnetic sorting and maintained in neurosphere conditions	Neurosphere medium: DMEM/F12, FGF2 (20 ng/mL), EGF (20 ng/mL), B27 (2%), L-Glut (2 mM)	Neurosphere-derived cultures: *ICC:* SOX10, P75	In vitro neural differentiation	[37]
	Direct isolation STRO-1^+ve^, c-KIT^+ve^, CD34^+ve^ magnetic sorting	a-MEM, 10% FBS, L-Glut (2 mM)	*ICC:* P75, SOX10, NES	In vitro neural differentiation (from standard FBS-containing cultures) and in vivo grafting for peripheral nerve repair (magnetic sorted cells)	[38]
	Monolayer: Cultures established in serum-containing medium and transferred to serum-free, MSC commercial medium (STP)	STP+NTP Medium: StemPro MSC SFM, BDNF (500 ng/mL), NT3 (20 ng/mL)	*RT-PCR:* Upregulation of HNK-1 and P75 *ICC:* P75	In vitro glial and neural differentiation	[39]
	Neurospheres: From monolayer cultures grown in serum-containing medium or serum-free medium	Monolayer (LAM coating)/Neurosphere medium: DMEM/F12, L-Glut (2 mM), EGF (20 ng/mL), FGF2 (20 ng/mL), IGF (50 ng/mL), B27 (2%), N2 (1%),with or without BMP4 (10 ng/mL)	Monolayer: *ICC:* NES, SLUG, SNAIL1, SOX9, P75. Neurospheres: *ICC:* NES, SOX2, SLUG, SNAIL1, SOX9. SOX10 and P75 (when supplemented with BMP4). *RT-PCR:* AP2a, P75, SNAIL1, SOX10 (when supplemented with BMP4)	In vitro neural differentiation	[40]
	Neurosphere Cultures grown in standard FBS-containing medium	Neurosphere: DMEM/F12, L-Glut (2 mM), EGF (20 ng/mL), FGF2 (20 ng/mL), heparin (5 ng/mL), B27 (1%), N2 (1%)	*RT-PCR:* AP2a, P75, NES, Sox2	In vitro differentiation into corneal endothelial-like cells	[41]

### 3.2. NCSCs Derived from Periodontal Ligament

The periodontal ligament (PDL), a connective tissue that allows tight and strong attachment of teeth to the maxillary and mandibular bone, also contains cells with mesenchymal and NCSC stem cell features [42]. Early evidence of NCSCs from PDL was obtained by culturing the cells in inductive medium containing chicken embryo extract, EGF and FGF2. These conditions yielded cells expressing *NES*, *P0*, *TUBIII*, *NFM* and *a-SMO* at the mRNA level, representing markers belonging to or derived from the NCSC phenotype. Additionally, an independent report also described a putative undifferentiated population of NSCSs from PDL, defined by HNK-1 and P75 markers present in <10% of the cells in culture [27]. On the other hand, Pelaez et al. [28] used magnetic beads to isolate CX43^+ve^ cells as a proposed NCSC population in PDL. This report showed that CX43^+ve^ cells showed greater expression of OCT4, NANOG, NCAD and SOX2 at the protein level, as well as higher expression of the relevant genes, in comparison to unfractionated or unsorted cell cultures. Importantly, the presence of SOX10 and P75 was identified in the positive fraction. The detection of pluripotent markers as well as the ability of CX43^+ve^ cells to form teratomas suggests a degree of pluripotency in NCSCs and highlights the importance of NCSC selection and isolation to achieve homogeneous cultures [28]. A more recent report by Ramírez-García et al. [29] provides a deep analysis of the effect of WNT activation and TGF-β inhibition for a PDL NCSC phenotype. Using flow cytometry, the authors showed a larger population of P75 and HNK-1 positive cells when these pathways were modified. Importantly, this phenotype was found to be dynamic and negatively affected by the presence of serum in the culture. The authors argue that the dynamism can be partially attributed to the cells’ capacity to undergo EMT or mesenchymal-to-epithelial transition (MET) as evidence of a NCSC phenotype [29]. Such dynamism and the impact of serum also provides evidence of the differences found in PDLs grown in MSC vs. NCSC conditions. Although PDL-derived NCSCs were defined by the expression of neural crest-related markers, their differentiation capability was untested. Thus, functional evidence is required as proof of concept for regenerative medicine.

### 3.3. NCSCs from Developing Teeth and the Adult Dental Pulp

The dental pulp is a well-documented source of adult stem cells. It consists of the soft tissue within a tooth and contains a variety of cells including vascular cells, fibroblasts, odontoblasts and stem cells [1,43]. The current literature describes dental pulp cells depending on their anatomical location as well as their developmental stage as described below.

#### 3.3.1. NCSCs Derived from the Dental Pulp of Exfoliated Deciduous Teeth (SHEDs)

Stem cells from human exfoliated deciduous teeth cells (SHEDs) have recently been investigated for the expression of neural crest markers [30]. The SHED phenotype can also be modified by the culture conditions. In a study analogous to their previous work on PDL-derived NCSCs [29], Gazarian and Ramírez-García [30] tested the effect of WNT pathway activation and TGF-β blockade as a growth condition for NCSCs. These pathway modifications provided a deeper insight of neural crest and mesenchymal marker dynamics. Flow cytometry data showed an increased population of cells expressing the neural crest markers P75^+ve^ and HNK-1^+ve^, and a decrease of the cells bearing the mesenchymal marker CD90^+ve^ in the neural crest medium [30]. Once more, the dynamism and influence of serum reflects the importance of culture conditions.

#### 3.3.2. NCSCs Derived from the Apical Pulp or Apical Papilla (SCAPs)

Stem cells from apical pulp or apical papilla (SCAPs) are often described separately from dental pulp stem cells (DPSCs) [described below] due to recognized differences in gene expression in vivo and in vitro [44]. Both SCAPs and DPSCs have been widely characterized as MSCs, but there is evidence of their NCSC phenotype. An early report described SCAPs-NCSCs with a migratory capacity to outgrow from tissue explants in vitro; when analysed by flow cytometry, these motile cells still presented typical MSC markers such as CD90, CD73 and CD166, but also expressed the neural crest markers *SNAI1*, *SNAI2*, *SOX9* and *TWIST* at the mRNA level [31].

Further evidence has been presented by growing SCAPs as neurospheres [32]. The expression of MSI1, P75 and NES proteins was shown by immunocytochemistry in the neurospheres, in addition to *MSI1*, *SLUG*, *SNAIL* and *P75* mRNA. The neurospheres also presented a reduced signal of the MSC markers CD166 and CD105 compared with the monolayer parental cultures [32]. In terms of their differentiation potential, NCSCs from SCAPs showed classical differentiation potential into adipogenic, myogenic, chondrogenic and also neurogenic fates. This report supports that MSC markers can be reduced when a NCSC phenotype is induced. Nevertheless, a relevant functional experiment is still lacking to support a specific application for SCAPs [32]. Recent work has demonstrated that self-forming spheres derived from SCAPs express HNK-1 and P75, providing further evidence that NCSCs can be obtained from SCAPs. These NCSCs were able to differentiate into an odontogenic phenotype which was enhanced by the addition of Platelet-Rich Plasma, but the results lack a functional test [33].

#### 3.3.3. NCSCs Derived from Dental Follicle

The dental follicle is part of the developing tooth and is defined as a loose connective tissue derived from the ectomesenchyme that surrounds the tooth germ [45]. Dental follicle stem cells have been obtained from developing third molars. In culture, a proportion of dental follicle cells expressed neural crest markers such as HNK-1, NES, P75 and SOX2 as observed by immunocytochemistry. These markers were observed in standard culture conditions that included a monolayer culture in 10% FBS [34].

#### 3.3.4. NCSCs Derived from the Dental Pulp

The term dental pulp stem cell refers to cell cultures derived largely from third molars but can include cultures from the pulp of other teeth. Cell cultures are obtained from the whole pulp tissue without distinguishing between different anatomical regions (such as the apical pulp) [1,43].

Early evidence of NCSCs from DPSCs came from Stevens et al., who first acknowledged NCSC characteristics in hDPSCs. NCSCs were described as a CD34^-ve^ population with label-retaining ability, and the capability of forming spheres and expressing P75 and NES. A report from Al-Zer et al. [35] presented evidence for the growth and expansion of cells migrating from dental pulp explants. Using a defined medium containing EGF and FGF2, the migratory cells were shown by immunocytochemistry to express P75, NES and SOX10, and were able to form spheres as well as be induced to osteogenic, glial and melanocytic differentiation [35].

Recently, a multistep protocol for the maintenance of the neural crest phenotype in human DPSCs was described, which revealed an enhanced hDPSC neurogenic ability in a subpopulation defined by STRO-1^+^/C-KIT^+^/CD34^+^ surface markers [46]. The same subpopulation showed an ability to improve peripheral nerve regeneration in an in vivo model of nerve graft repair [38]. A follow-up study by the same group suggested that the described subpopulation could maintain a neural crest phenotype by culturing in 3D (floating sphere) conditions [37]. Importantly, sphere-derived cultures not only expressed the neural crest markers P75 and SOX10 but were also able to differentiate into neural-like cells, with recognizable Na^+^ and K^+^ currents. Although no action potentials were detected, this study provided the first functional evidence from dental pulp cells characterized as NCSCs.

A recently published approach coupled the use of a commercial serum-free mesenchymal stem cell medium with the neurotrophic factors NT3 and BDNF [39]. Noteworthily, the combination of these factors is usually employed for neural differentiation. Nevertheless, the authors showed an upregulation of the neural crest-related markers HNK-1 and P75, as well as a more efficient differentiation into glial and neuronal phenotypes from cultures supplemented with NT3 and BDNF [39]. Similar to other reports, the cultures were established in serum-containing conditions before being transferred to serum-free media.

A publication from our group explored in more detail the NCSC phenotype in human dental pulp cells as monolayer and as neurosphere cultures [40]. Contrary to most reports, the tissue was established from the beginning into neurogenic, serum-free culture conditions. Interestingly, cells grown as monolayer in serum-rich and serum-free conditions all presented a basal expression of the NCSC-related markers SLUG, SNAIL1, NES, SOX9 and P75 as shown by IHC. There was no difference in *SOX2*, *NES* and *P75* mRNA expression between serum-rich and serum-free monolayer conditions. However, by culturing the cells as neurospheres, the NCSC signature was more evident in the cells established in serum-free cultures. The NCSC phenotype could be further enhanced in the presence of BMP4. This signature was described by an upregulation of *AP2a*, *SNAIL1*, *P75 and SOX10* mRNA. Additionally, it was found that a neural-like differentiation was more evident in cultures from serum-free conditions supplemented with BMP4 as judged by a more defined morphological change and the upregulation of *GAP43*, *NFH* and *SYN1* mRNA. The mentioned report presented a broader characterization of the NCSC phenotype in human dental pulp cells, and also supported how 3D culturing is important to enhance a NCSC signature and downstream differentiation to neural-like cells [40].

Facilitation of NCSC derivation via neurosphere formation was further supported in a report showing a higher relative expression of *P75*, *SOX2*, *NES* and *AP2* on day 4 of sphere formation. The expression levels of these markers were significantly reduced after culturing them back into monolayer conditions as determined by RT-qPCR [41]. Importantly, the authors also showed that NCSCs from the dental pulp were able to differentiate into corneal endothelial-like cells, indicating efforts toward a specialized application [41].

## 4. Considerations of Growth Conditions and Strategies for Isolation

Previous work has used a variety of different techniques and culture conditions when characterizing NCSCs. It is of particular importance to highlight these different strategies to be able to pinpoint a particular method that would allow NCSCs to be obtained in a standardized and reproducible way. The following methods include growing conditions depleted of animal serum and highlight components that could be replaced by xeno-free factors for future clinical-grade application, and therefore more realistic therapeutic scenarios [47,48].

### 4.1. EGF/FGF2 Containing Media

**Figure 2:**
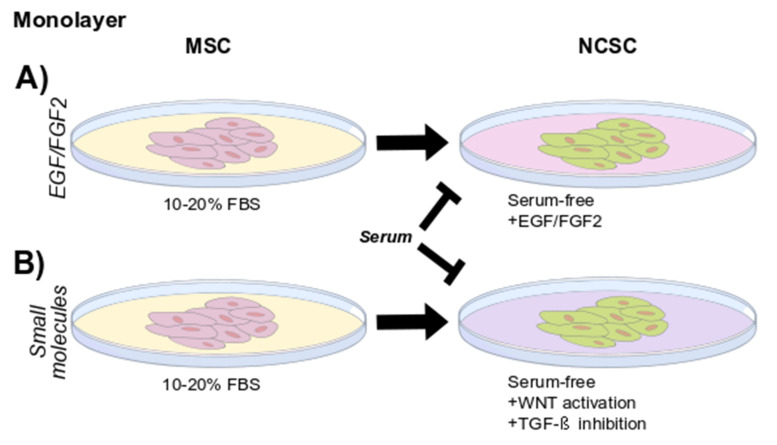
Isolation and growth of NCSCs from DRSCs in monolayer cultures: After initial culture in FBS, NCSCs can be induced or enriched by modifying the growth medium to serum-free conditions together with (**A**) growth factors such as EGF and FGF2 and/or (**B**) small molecules such as WNT activators and TGF-β inhibitors.

Another strategy to obtain NCSCs is to use standard serum-containing conditions in initial culturing and then change these conditions to serum-free media supplemented with EGF and FGF2 in a neurobasal solution. This approach has been used for gingiva and periodontal ligament derived NCSCs (Figure 2A) [26,27]. These factors have been used to maintain neural stem or progenitor cells in other models and are usually needed in multistep neural differentiation protocols as seen in protocols where DRSCs were originally cultured as MSCs [49]. Together, EGF and FGF2 have been found to allow proliferation while maintaining neural commitment [14].

### 4.2. Small Molecules

Periodontal ligament, deciduous teeth and gingival cultures grown under WNT activation and TGF-β inhibition present a NCSC phenotype (Figure 2B) [26,29,30]. This approach resembles a developmental path that has been used to derive neural crest cells from human pluripotent stem cells [50]. Furthermore, the plasticity shown by the cells in culture—which were able to dynamically respond to the cues in the medium, particularly serum concentrations—provides further evidence of a neural crest resemblance. Characterization of cells in this condition has provided evidence of the effect of serum in the culture on the NCSC phenotype.

### 4.3. Neurosphere Formation

**Figure 3:**
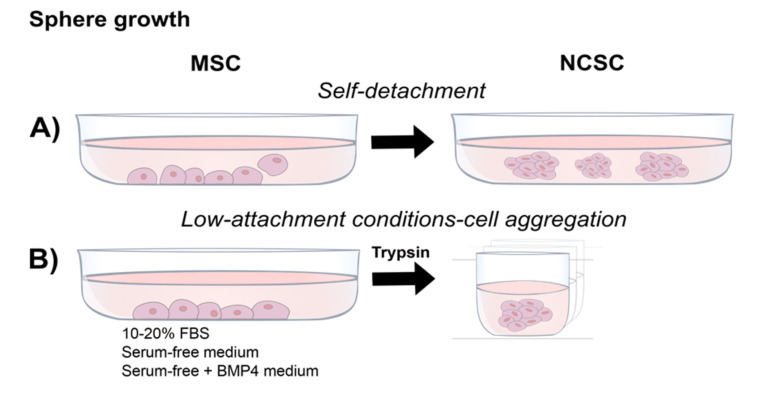
Isolation and growth of NCSCs from DRSCs by sphere formation: NCSCs can be induced or enriched by growing the cultures in serum-free conditions in low-attachment conditions, (**A**) promoting the formation of free-floating spheres by self-detachment, or (**B**) by forcing cell aggregation in spheroid cultures.

A modification of cultures containing EGF and FGF2 is to use low-attachment conditions to grow the cells as neurospheres or 3D cultures (Figure 3). The ability to form spheres expressing relevant NCSC markers has resulted in a common test for a neural crest phenotype. When compared, NCSCs from P0/WNT1-CRE mouse tissues, selected by enhanced green fluorescent protein (EGFP) expression, showed a significantly higher ability to form spheres than did EGFP^-ve^ cells, suggesting an enhanced capacity intrinsic to NCSCs to grow in this state [12]. Results from human gingival and apical pulp-derived NCSCs suggests that neurosphere formation increased NCSC markers and decreased mesenchymal markers, indicating a molecular resemblance to a neural crest phenotype [25,32]. Notably, some authors describe an intrinsic ability of the monolayer cells to detach from the surface (Figure 3A; [26]) while others grow the cells directly in suspension in low-attachment culture wells (Figure 3B; [37,40]).

### 4.4. Direct Isolation (Cell Sorting)

**Figure 4:**
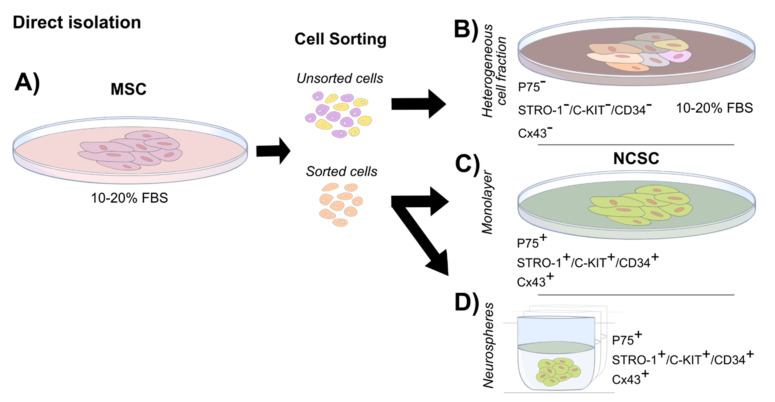
Isolation and growth of NCSCs from DRSCs by direct isolation: NCSCs can isolated by cell sorting from heterogeneous serum-rich cultures. (**A**) The identification of specific surface markers allows their use to sort for a particular cell fraction with NCSC characteristics (sorted cells) and a fraction of cells negative to those markers (unsorted cells) (**B**). The unsorted fraction can contain a variety of subpopulations. Common downstream applications of the sorted NCSCs can be performed as monolayer (**C**) or maintained as NCSCs as neurospheres (**D**).

Using cell sorting would allow the enrichment of a specific NCSC fraction from cells grown in MSC conditions in monolayer (Figure 4A). This selection step results in different sub-populations of cells not selected by a particular marker (Figure 4B) and cells expressing a marker of interest (Figure 4C,D). In this regard, P75 and HNK-1 are natural candidates as the observations from studies on PDLs and SHEDs indicate them as potential markers for cell sorting [29]. It can be hypothesized that cell sorting for these markers could present similar characteristics to Cx43+ve cells in PDL [28]. In this regard, it must be noted that the use of a single marker might still select a heterogeneous cell population. A double or multiple marker selection may be necessary in order to extract relevant cell populations. As an example, differentiation into mesenchymal lineages using P75 as the sole marker for direct isolation has given contradictory results in two independent studies from exfoliated deciduous teeth and dental pulp cells [51,52]. Although differences might be a result of the cell origin and methodological discrepancies, it also suggests that a single marker might not be robust enough to select a homogeneous cell fraction. To support this, Pan et al. [53] characterized the P75 fraction from DPSCs, arguing toward the possibility of it being a neurogenic fraction due to the co-localization of neural factors such as SOX1, NES and SOX2. Furthermore, the recent characterization and functional evidence provided from a STRO-1+/C-KIT+/CD34+ sorted subpopulation obtained from dental pulp has proven adequate for NCSC isolation [38]. Interestingly, a direct isolation combined with neurosphere growth has been proposed as an improved method (Figure 4D). Combining the direct isolation of a STRO-1+/C-KIT+/CD34+ subpopulation with neurosphere growth has shown that a greater neurogenic potential could be achieved. This observation was supported by the expression of the neuronal markers MAP2 and TUBIII as well as sodium and potassium currents; notably, no action potentials were detected [37].

## 5. Important Considerations from Previous Reports of DRSCs Characterized as MSCs

The first reports on DRSCs focused on characterizing human DRSCs as mesenchymal stromal cells expressing CD105, CD90, STRO-1 among others [1,23,42,54]. Attempts to directly differentiate dental MSCs into neural lineages have mostly failed to show a mature or functional phenotype when grown from their basal conditions in media-containing serum. Indeed, careful analysis of previous reports indicates that mature neural induction is achieved only after multistep differentiation protocols including EGF/FGF2 conditioning, epigenetic modification and neurosphere formation [49,55,56]. This suggests that successful and physiological relevant differentiation is achieved when a putative neurogenic population (e.g., NCSCs) is induced or selected as an intermediate step. Thus, ablating the MSC phenotype, as observed in different DRSCs, could be a requisite for successful neural maturation [26,30,32]. Notably, a report has revealed that only 8.3% of DPSCs and 21.2% of gingiva-derived mesenchymal stem cells were able to acquire a mature phenotype capable of firing action potentials using an established protocol for neural differentiation [57]. This report highlights the limitations even of successful protocols.

DRSCs described as MSCs have also appeared in preclinically relevant reports as evidence of their translational use in regenerative medicine. For instance, Sakai et al. [58] provided key evidence of the potential of SHEDs and DPSCs to replace lost oligodendrocytes after spinal cord injury in vivo. In another key report, Fujii et al. [59] generated dopaminergic neurons from deciduous teeth and tested them in vivo in a rat model of Parkinson’s disease; the results supported a beneficial paracrine effect by the grafted SHEDs [59]. In a separate example, Martens et al. [60] differentiated dental pulp cells into Schwann cells that, when inserted in artificial conduits for nerve regeneration, allowed myelin formation and axon guidance in rats. A recent publication also provided evidence of the beneficial trophic support of dental pulp in an in vitro organotypic hippocampal model [61]. It should be noted that the neurogenic and translational potential from MSC-described DRSCs is defined largely by their neurotrophic, glial-like support rather than direct neuronal replacement. Additionally, DPSCs have recently been shown to differentiate into auditory-like neurons when co-cultured with rat auditory brain stem, providing pioneering evidence of DPSC differentiation associated with a host tissue [62].

Nevertheless, few translational examples from DRSCs described as NCSCs have been described. In DRSC-NCSCs, such potential is commonly assumed, and remains largely unproven, with the exception of gingiva and DPSC in peripheral nerve regeneration [26,38]. However, evidence is lacking for specific applications in which DRSCs could replace damaged or lost neurons.

## 6. Conclusions and Considerations for Future Applications

As described above, DRSCs can be induced to a NCSC phenotype and hold promise to be used in regenerative medicine. However, current reports have been largely limited to only characterizing the NCSC phenotype rather than testing their functional behaviour, with only a few examples of physiological tests in nerve regeneration [26].

Reports of cultures initially treated and characterized as MSCs in standard serum-containing media have provided evidence of their great potential. For example, the use of DRSCs (described as MSCs) has been proposed in spinal cord regeneration, peripheral nerve repair, neurodegenerative disorders (e.g., Parkinson’s disease), hearing loss, and corneal endothelial therapies [58,59,60,61,62]. Nonetheless, further research is needed to overcome the limitations of these studies, such as understanding the mechanism of action (e.g., cell replacement vs immunomodulation), lack of in vivo testing, and inadequate culture conditions and scalability.

The initial growing conditions used to produce cells for clinical use are critical. Many of the protocols described in the literature to date would not be translatable to a therapeutic scenario, due to the presence of factors from animal origin, which do not comply with good clinical practice (GCP) standards. Therefore, future work would benefit from developing approaches that use serum-free or GMP-compliant media in initial derivation and expansion conditions. Critically, these conditions have been shown to be compatible with NCSC-DRSC derivation and maintenance [40,48]. Evidence of a molecular neural crest signature together with functional efficacy in relevant regenerative models are also required to advance the field. Future research should be encouraged to use xeno-free medium to establish NCSCs cultures and test their properties in functional, clinically-relevant models to close the gap toward the use of DRSC for clinical applications.

## Abbreviations

AP2A	Activator Protein 2a
BDNF	Brain-Derived Neurotrophic Factor
ΒME	2-Mercaptoethanol
BMP4	Bone Morphogenetic Protein 4
COL1	Collagen Type I
CX43	Connexin 43
DLX6	Distal-Less Homeobox 6
DMEM	Dulbecco’s Modified Eagle Medium
DPSC	Dental Pulp Stem Cell
DRSCS	Dental-Related Stem Cells
E-CAD/CDH1	E-Cadherin
EGF	Epidermal Growth Factor
EMT	Epithelial to Mesenchymal Transition
FBS	Fetal Bovine Serum
FGF	Fibroblast Growth Factor
FN	Fibronectin
FOXD3	Forkhead Box D3
GAP-43	Growth Associated Protein 43
GCP	Good Clinical Practice
GFP	Green Fluorescent Protein
GMP	Good Manufacturing Practice
HDPSC	Human Dental Pulp Stem Cell
HNK-1	Human Natural Killer-1
ICC	Immunocytochemistry
IFG	Insulin Growth Factor
LAM	Laminin
MART-1	Melanoma Antigen Recognized by T-Cells
MEM	Minimum Essential Medium
MET	Mesenchymal to Mesenchymal Transition
MSCS	Mesenchymal Stromal Cells
MSI1	Mushashi-1
MSX2	Msh Homeobox 2
NANOG	Nanog Homeobox
NCAD	N-Cadherin
NCC	Neural Crest Cells
NCSC	Neural Crest-Derived Stem Cell
NCSCM	Neural Crest-Derived Stem Cell Medium
NES	Nestin
NF	Neurofilament
NFH	Neurofilament-High
NFM	Neurofilament-Medium
NT3	Neurotrophin-3
OCT4	Octamer-Binding Transcription Factor 4
P75	Low-Affinity Nerve Growth Factor Receptor
PAX3	Paired Box 3
PDL	Periodontal Ligament
PLO	Poly-Ornithine
RT-QPCR	Retro-Transcriptase Quantitative Polymerase Chain Reaction
SCAP	Stem Cells from Apical Pulp
SHED	Stem Cells from Human Exfoliated Deciduous Teeth
SLUG	Snail Family Transcriptional Repressor 2
SNAIL	Snail Family Transcriptional Repressor
SOX2	Sry-Box Transcription Factor 2
SOX9	Sry-Box Transcription Factor 9
SOX10	Sry-Box Transcription Factor 10
SYN	Synapsin
TGF-Β	Transforming Growth Factor Beta
TUBIII	Class III Β-Tubulin
TWIST	Twist Family Bhlh Transcription Factor
VIM	Vimentin
WNT1	Wingless-Related Integration Site-1
ZEB	Zinc Finger E-Box Binding Homeobox
